# Comparative observational study of mortality amenable by health policy and care between rural and urban Finland: no excess segregation of mortality in the capital despite its increasing residential differentiation

**DOI:** 10.1186/s12939-016-0348-2

**Published:** 2016-04-05

**Authors:** Markku Lehikoinen, Martti Arffman, Kristiina Manderbacka, Marko Elovainio, Ilmo Keskimäki

**Affiliations:** Department of Social Services and Health Care, City of Helsinki, P.O. Box 6100, 00099 Helsinki, Finland; Network of Academic Health Centres and Department of General Practice and Primary Health Care, University of Helsinki, P.O. Box 20 , Tukholmankatu 8 B, 00014 Helsinki, Finland; Department of Health and Social Care Systems, Social and Health Systems Research Unit, National Institute for Health and Welfare (THL), P.O. Box 30, 00271 Helsinki, Finland; Institute of Behavioural Sciences, University of Helsinki, P.O. Box 9, 00014 Helsinki, Finland; School of Health Sciences, University of Tampere, 33014 Tampere, Finland

**Keywords:** Health inequalities, Residential differentiation, Positive discrimination, Social mixing, Register data

## Abstract

**Background:**

Large cities are often claimed to display more distinct geographical and socioeconomic health inequalities than other areas due to increasing residential differentiation. Our aim was to assess whether geographical inequalities in mortality within the capital (City of Helsinki) both exceeded that in other types of geographical areas in Finland, and whether those differences were dependent on socioeconomic inequalities.

**Methods:**

We analysed the inequality of distribution separately for overall, ischemic heart disease and alcohol-related mortality, and mortality amenable (AM) to health care interventions in 1992–2008 in three types of geographical areas in Finland: City of Helsinki, other large cities, and small towns and rural areas. Mortality data were acquired as secondary data from the Causes of Death statistics from Statistics Finland. The assessment of changing geographical differences over time, that is geographical inequalities, was performed using Gini coefficients. As some of these differences might arise from socioeconomic factors, we assessed socioeconomic differences with concentration indices in parallel to an analysis of geographical differences. To conclude the analysis, we compared the changes over time of these inequalities between the three geographical areas.

**Results:**

While mortality rates mainly decreased, alcohol-related mortality in the lowest income quintile increased. Statistically significant differences over time were found in all mortality groups, varying between geographical areas. Socioeconomic differences existed in all mortality groups and geographical areas. In the study period, geographical differences in mortality remained relatively stable but income differences increased substantially. For instance, the values of concentration indices for AM changed by 54 % in men (*p* < 0.027) and by 62 % in women (*p* < 0.016). Only slight differences existed in the time trends of Gini or in the concentration indices between the geographical areas.

**Conclusions:**

No geographical or income-related differences in the distribution of mortality existed between Helsinki and other urban or rural areas of Finland. This suggests that the effect of increasing residential differentiation in the capital may have been mitigated by the policies of positive discrimination and social mixing. One of the main reasons for the increase in health inequalities was growth of alcohol-related mortality, especially among those with the lowest incomes.

## Background

Despite the long-term health policy goals underlying equity in health and health services, both geographical and socioeconomic health inequalities have increased in Finland, including in the City of Helsinki, which is the capital of Finland and forms the main part of the Helsinki Metropolitan Area [1, 2]. In political debate, Helsinki and its metropolitan area are often considered as a special case due to both the greater number of migrants than elsewhere in Finland [3] and the accumulation of social problems e.g. homelessness, use of alcohol, and in the case of Helsinki, child poverty [4–6]. The Helsinki Metropolitan Area also has an increasing level of segregation, although it is still fairly low by international standards [7–9]. Thus a more appropriate term in the Finnish context is residential differentiation.

The main body of studies linking segregation to health and health care usage originates from the USA, and concentrates on racial disparities. These disparities contribute to socioeconomic inequality and push disadvantaged ethnic groups to areas with poor access to care. Thus, both individual and community level factors have a role in the developments that link segregation to polarization of health problems [10–13]. Four possible pathways are suggested to mediate this association: through individual socioeconomic status, physical and social hazards of neighbourhoods, social capital, and individual risk behaviour [14]. Across Europe this association is not straightforward, and remains thus understudied; even a comparison of ethnic segregation is complicated, as minorities, public services, collected data, and area definitions vary between countries [15, 16]. However, worse self-reported health by ethnic minorities and migrants compared to the majority population in Europe [17] suggests geographical accumulation of health risks with ethnic segregation. We found only one paper that assessed the association between socioeconomic residential segregation and health in Europe [18]. It suggests that residential segregation by wealth partly mediates the effect of income inequality on acute myocardial infarction. The increase of socioeconomic residential segregation within European capitals [15], though still below that seen in the other continents, emphasizes the importance of this finding. Despite these observations, the effect of increasing segregation on health inequalities between a metropolis and other types of geographical areas within one country remains unclear. Thus, an analysis of health-related endpoints in a European metropolis with increasing segregation is of interest.

In this study, we evaluate whether residential differentiation within Helsinki manifests itself as geographical health inequalities not displayed by other parts of the country; a phenomenon that we refer to as the ‘metropolitan effect’. To measure the impact of increasing segregation on health inequalities, we chose to observe and compare changes in overall, ischemic heart disease (IHD), and alcohol-related mortality and mortality amenable to health care interventions in three types of geographical areas.

Mortality amenable to health care interventions (hereafter *amenable mortality*) is a compilation of conditions preventable by well-functioning health care. Thus it is an instrument for assessing health care quality over time, and highlighting flaws in the arrangement of care. Analysis of these flaws, however, necessitates more elaborate methods [19]. Throughout Finland, amenable mortality has declined more rapidly than overall mortality. This decline differs between counties, as well as between the major districts of Helsinki, with increasing geographical polarization [20, 21]. Recent studies of Helsinki concentrate on life expectancy and disease prevalence [1, 2]. While it has been suggested that part of IHD mortality should be attributed to health care, we excluded IHD mortality from amenable mortality, since along with alcohol-related mortality (deaths both directly caused by alcohol and to diseases related to alcohol abuse) it expresses also lifestyle-related deaths preventable by health policy [22, 23].

Our hypothesis was that if the metropolitan effect exists in Helsinki, the geographical health inequalities of the capital should exceed those of the other types of geographical areas, and the level of amenable mortality in it should decrease more slowly than elsewhere. In addition, we assume that due to the metropolitan effect, mortality differences between socioeconomic groups in Helsinki would exceed those of the other types of geographical areas in Finland.

### City of Helsinki

Finland experienced a deep economic recession in the early 1990s, which in Helsinki boosted residential differentiation. First a direct reaction to economic difficulties increased unemployment. Then later on rapid economic growth, driven by the development of the information technology industry, resulted in growing incomes among the well-off [24]. At present, social disadvantages, such as unemployment, poverty, and alcohol and substance abuse, are concentrated along the railway and underground network as well as both the eastern and north-eastern parts of the city. As the majority of Helsinki’s migrant population reside in these very same neighbourhoods [25], both socioeconomic and rapidly increasing ethnic differentiation seem to relate to the same reasons: social housing’s abundant housing stock and neighbourhood location [26]. However, the ethnic differentiation in Helsinki is not as pronounced as elsewhere, such as in the Swedish metropolitan areas, where peak segregation occurs between the native and the migrant populations [27]. While the proportion of migrants in the inner City of Helsinki and Stockholm are alike, in outer Stockholm it is, at its highest, almost double that of Helsinki.

In its housing policy, Helsinki has attempted to support social mixing by being actively involved in building social housing. This is in contrast for instance to the policies in Stockholm, where social housing schemes have been privatised [28, 29]. In addition, Helsinki has implemented positive discrimination (PD) in its public services. Since the 1990s additional resources have been targeted to health care in neighbourhoods with more challenging socio-demographic structures [30]. Despite these measures, wealthy and educated people have selectively chosen not to settle in disadvantaged areas [31]. Furthermore, a similar phenomenon within the school system has embedded the differentiation into future society. In fact, the ethnic differentiation within the school system is already more intense than that observed between the neighbourhoods [32, 33]. Altogether, the recent residential differentiation in the Helsinki Metropolitan Area, in regards to both social problems and ethnic groups, follows the increasing international trend seen in other metropolitan areas [8, 25]. Elsewhere in Finland this differentiation remains under-studied: no countrywide evidence about its level exists. However, in the city of Turku, the third largest urban area of Finland, differentiation resembles that of Helsinki [34].

Our objective was to assess whether the above-described metropolitan effect exists in the City of Helsinki, i.e. did the capital city have an excess of geographical inequality in mortality — unrelated to socioeconomic differences — compared to other types of geographic areas?

## Methods

The analyses focused on overall, amenable, IHD, and alcohol-related mortality. We divided Finland into three types of geographical area: 1) City of Helsinki (HKI), 2) nine of the next most-populated municipalities (NNMM), and 3) the rest of Finland (RoF), including smaller towns and rural areas. Further, we divided these geographical areas into small areas roughly representing districts served by a single health care provider unit. In HKI and NNMM, we used municipal administrative areas, which are also usually defined as responsibility areas for GP-led primary care health centres. In RoF, the small areas were defined according to the municipal health authorities, e.g. a single municipality or a consolidation of tiny municipalities. The number of residents between small areas somewhat varied, for example, in 2008: 3633–23 105 in HKI, 3255–39 723 in NNMM, and 3056–38 151 in RoF. Except for the first time period 1992–94, usage of biannual time periods enabled enough cases for the analyses of small areas.

The Causes of Death statistics provided by Statistics Finland describe mortality data for all deaths in the Finnish population aged 25–74 between 1992 and 2008 (Table 1). The resident population aged 25–74 formed the population at risk. We based the classification of deaths amenable to health care interventions on the Nolte and McKee list, and adapted it according to the Australian & New Zealand Atlas of Amenable Mortality [19, 35]. Thus we complemented the list with: 1) infections that are preventable by vaccination and other public health measures, asthma, and COPD, and 2) benign tumours and malignant neoplasm of the bladder. The afore-mentioned diagnoses can be both preventable and effectively treated, especially in younger age groups, while the latter are suggested as mainly treatable [35]. For exact diagnoses codes we used the list published by Lumme et al. [36]. The study period covered the transition from ICD9 to ICD10 in 1996.

**Table 1:** Number (N) of deaths and proportions of examined mortalities in the study population

	1992			2008		
	HKI	NNMM	RoF	HKI	NNMM	RoF
N	2008	3869	14 377	1757	3553	11 682
AM	19.9 %	19.7 %	19.4 %	17.1 %	17.8 %	16.3 %
IHD	23.0 %	26.6 %	30.4 %	16.2 %	15.9 %	18.5 %
AR	7.2 %	6.3 %	4.0 %	12.9 %	11.9 %	11.0 %

*HKI* City of Helsinki, *NNMM* nine next-most populated municipalities, *RoF* rest of Finland, *AM* amenable mortality, *IHD* ischemic heart disease mortality, *AR* alcohol related mortality

Individual-level register data for annual employment statistics maintained by Statistics Finland provided the indicators for gender, age, living arrangements, area of residence, and income. Data on income came from annual tax registries. Age was classified into 5-year age bands, and income was adjusted for family size using the OECD equivalence scale [37]. Further, the population aged 25–74 was grouped into income quintiles according to quintile limits derived from the total Finnish population (Table 2). The lowest quintile covered also those with no recorded income (approximately 1–2 % of the total Finnish population). Analyses excluded the long-term institutionalized population, since the registers could not reliably provide data on their socioeconomic factors and living conditions. Personal identity numbers linked socioeconomic data to mortality data. Authorized personnel from Statistics Finland undertook the data linkages and the research group received anonymised data tabulated annually by sociodemographic variables to prevent indirect identification of individuals. Ethical consent for the study was received from the Ethical Review Board of the National Institute for Health and Welfare (THL) and permissions to use the data were received from THL and Statistics Finland. The analyses were performed separately for men and women, since earlier research suggested differential mortality patterns, larger socioeconomic mortality differences and higher overall mortality rates among men [38].

**Table 2:** Study population characteristics

		1992	2008
Study population		3 104 684	3 290 739
Gender	Men	48.8 %	49.6 %
	Women	51.2 %	50.4 %
Age group	25–39 years	36.7 %	29.3 %
	40–44 years	13.7 %	11.1 %
	45–49 years	11.5 %	11.2 %
	50–54 years	9.3 %	11.5 %
	55–59 years	8.0 %	12.0 %
	60–64 years	8.1 %	10.9 %
	65–69 years	7.2 %	7.6 %
	70–74 years	5.5 %	6.4 %
Geographical area	HKI	10.4 %	11.1 %
	NNMM	22.1 %	24.6 %
	RoF	67.5 %	64.3 %
Income quintile	Lowest	15.5 %	14.6 %
	2	18.4 %	17.1 %
	3	19.5 %	19.5 %
	4	21.7 %	22.7 %
	Highest	25.0 %	26.1 %
Living alone	Yes	15.9 %	21.6 %
	No	84.1 %	78.4 %
Long-term unemployed	Yes	1.4 %	2.5 %
	No	98.6 %	97.5 %

*HKI* city of Helsinki, *NNMM* nine next-most populated municipalities, *RoF* rest of Finland

The direct method of standardization was used to calculate separate age-standardized mortality rates for men and women in eight time periods. Gini coefficients (Ginis) were calculated to quantify the degree of geographical variation in mortality between small areas within each of the three regions. We ranked small areas by their relative mortality rates and computed Ginis to study the distribution of mortality in the three regions. Respectively, concentration indices (Cs) were calculated to highlight the income inequalities in mortality in the three geographical areas by ranking the tabulated data by income quintiles. We calculated age-standardized Ginis and Cs separately for men and women in eight time periods using the approach presented for the tabulated data by Kakwani et al. [39] and Doorslaer et al. [40] and further developed by Lumme et al. [36]. In addition, we calculated age- and income-standardized Ginis in order to evaluate the impact of socioeconomic factors in the small area variation of mortality. A concentration index value of 0 expresses absence of income-related inequality, a value of -1 absolute inequality in favour of the highest income quintile, and a value of 1 absolute inequality in favour of the lowest quintile. Gini values vary between 0 with absolute equality between small areas and 1 with all mortality concentrated in one small area.

We applied the Monte Carlo approach presented by Lumme et al. [36] in 2012 to estimate accurate Gini and C confidence intervals by replicating the estimation 300 times to account for the uncertainty. However, in the case of the Ginis for women’s alcohol-related mortality, confidence intervals without bootstrapping are presented, as bootstrapping proved unstable due to the low numbers of alcohol-related deaths among women. We estimated linear trends from linear regression models for Ginis and Cs to illustrate changes in the socioeconomic and small area distribution of mortality. In these analyses, usage of the inverse of the standard errors as weights accounted for the uncertainty. We evaluated the significance of unemployment and living alone for income differences in overall mortality by including them in age- and time-period-adjusted repeated-measures Poisson regression models, which were performed separately for men and women in the three types of geographical areas. We performed statistical analyses with the SAS system for Windows, release version 9.3 (SAS Institute, Cary, NC).

## Results

**Fig. 1:**
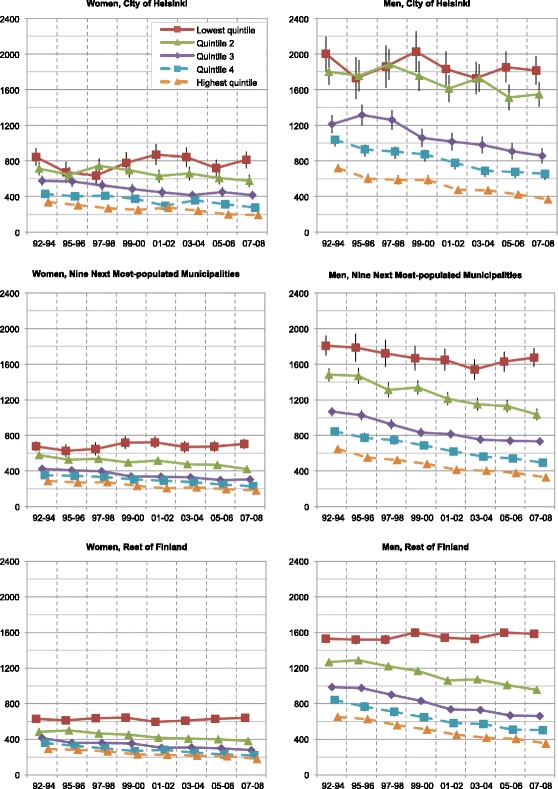
Age-standardized overall mortality rates of income quintiles in Finland per 100 000 population

**Fig. 2:**
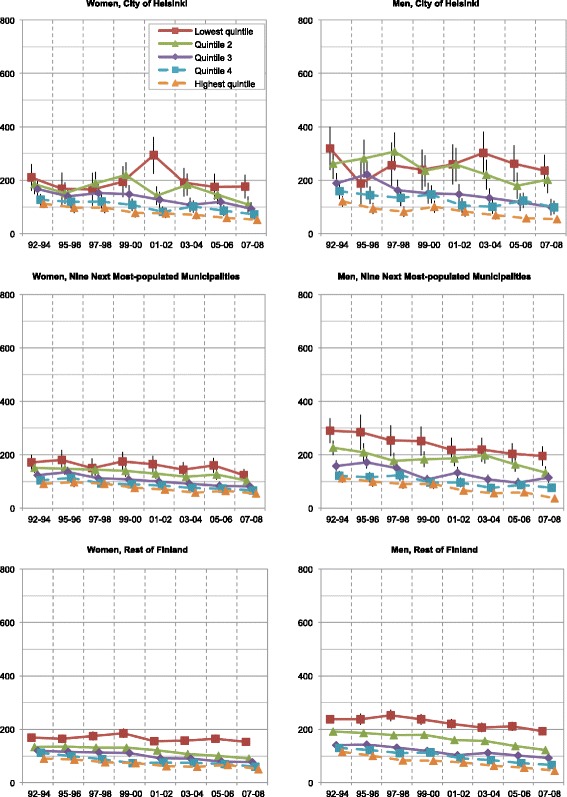
Age-standardized amenable mortality rates of income quintiles in Finland per 100 000 population

**Fig. 3:**
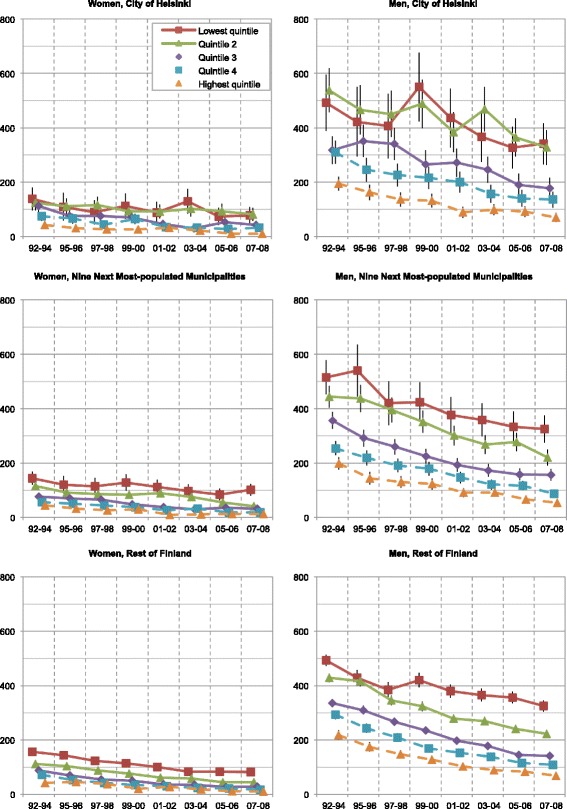
Age-standardized ischemic heart disease mortality rates of income quintiles in Finland per 100 000 population

Mortality rates of Finns aged 25–74 decreased in the period 1992–2008: 35–42 % for amenable mortality, 47–66 % for IHD mortality, and 24–32 % for overall mortality (Figs. 1, 2, and 3). The mortality rate ratios (RR) between men and women expressed higher mortality rates for men both throughout the country and the study period overall (RR 1.7–2.9) and IHD (RR 2.8–10.1) and alcohol-related mortality (RR 1.8–8.0). Corresponding amenable mortality rates showed only minor differences between genders (RR 0.7–1.9). Only at the end of the study period in the highest quintile did the RRs of amenable mortality slightly favour men.

**Fig. 4:**
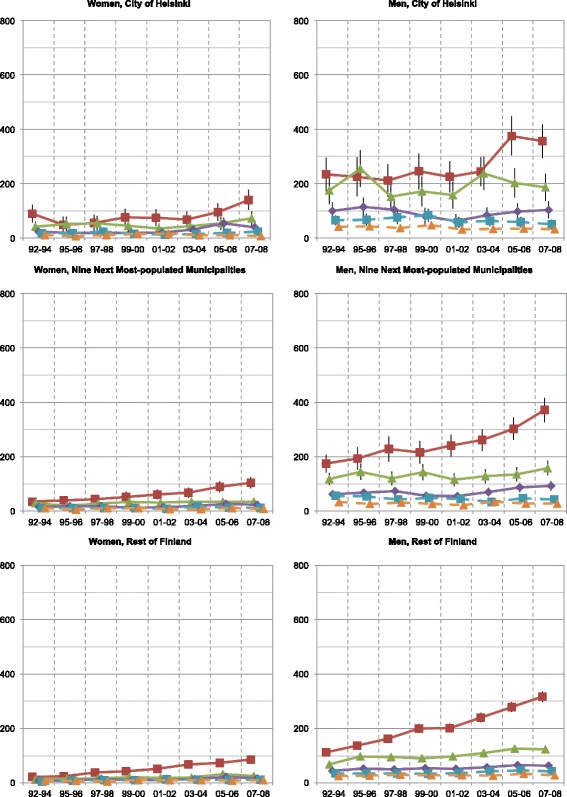
Age-standardized alcohol related mortality rates of income quintiles in Finland per 100 000 population

**Fig. 5:**
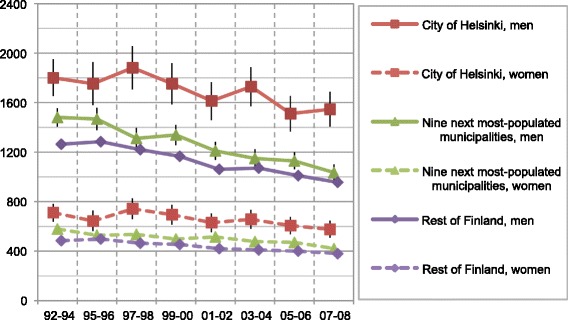
Age-standardized overall mortality rates of second lowest income quintile in Finland per 100 000 population

However, the observed decrease did not affect the overall mortality in the lowest income quintile, whose mortality remained stagnant or even increased throughout the country. Unlike in other mortality groups, alcohol-related mortality (Fig. 4) in the lowest quintile (Q1) increased dramatically (52–291 %). Some differences between the three geographical areas existed: the overall mortality rates in the second lowest quintile (Q2) in Helsinki slightly exceeded those of the other geographical areas (Fig. 5). To a lesser extent this phenomenon involved the second highest quintile (Q4) in women, and both the middle and the second highest quintiles (Q3–Q4) in men. The rates of the highest quintiles were similar throughout the country.

Baseline differences between geographical areas were almost non-existent (data not shown), except for the higher Ginis of alcohol-related mortality in women for the rest of Finland than elsewhere (0.17–0.24, Cl95 % 0.04 to 0.36, *p* = 0.013 to <0.001). Linear trends between the geographical areas were surprisingly consistent: only slight differences existed in single comparisons of Cs (data not shown). Thus, in the period 2007–2008, only the Ginis of alcohol-related mortality for women in the rest of Finland were elevated when compared to the other geographical areas.

To further investigate the geographical differences observed in the second lowest quintile, we analysed the effects of both long-term unemployment and living alone on overall mortality rates with additional Poisson regression models (Table 3). When comparing these models, the impact of living alone, measured by the change of risk ratios within all the income quintiles, was significant throughout the country. Long-term unemployment, however, had no significance in any of the three types of geographical areas. Overall, the decrease in mortality rates centred on the lowest quintile, though in Helsinki, among men, the decrease in risk ratios was more similar in both the lowest and the second lowest quintiles. In the three higher quintiles (Q3–Q5) the risk ratios were similar between the two models (data not shown).

**Table 3:** Risk ratios of overall mortality in two lowest income quintiles compared to the highest quintile

Quintile	Area	Men				Women			
		Model 1		Model 2		Model 1		Model 2	
		RR	95 % Cl	RR	95 % Cl	RR	95 % Cl	RR	95 % Cl
Q1	HKI	4.02	3.04–5.30	3.41	2.86–4.06	3.33	2.69–4.12	3.19	2.60–3.92
	NNMM	4.32	3.14–5.94	3.52	2.91–4.26	3.27	2.69–3.98	3.11	2.58–3.74
	RoF	3.46	2.57–4.66	2.95	2.52–3.46	2.82	2.30–3.45	2.72	2.25–3.28
Q2	HKI	3.24	2.47–4.25	2.84	2.33–3.46	2.50	1.99–3.15	2.37	1.88–3.00
	NNMM	2.83	2.09–3.84	2.56	2.09–3.14	2.23	1.85–2.69	2.13	1.78–2.55
	RoF	2.42	1.85–3.17	2.29	1.93–2.70	1.98	1.66–2.36	1.95	1.64–2.31

*HKI* City of Helsinki, *NNMM* nine next-most populated municipalities, *RoF* rest of Finland, *Model 1* no explanatory factors, *Model 2* long-term unemployment (not significant in any of the three regions) and living alone (men: *p* < .001 in all the three regions – women: *p* < .01 in HKI, and *p* < .001 in both NNMM and RoF)

**Fig. 6:**
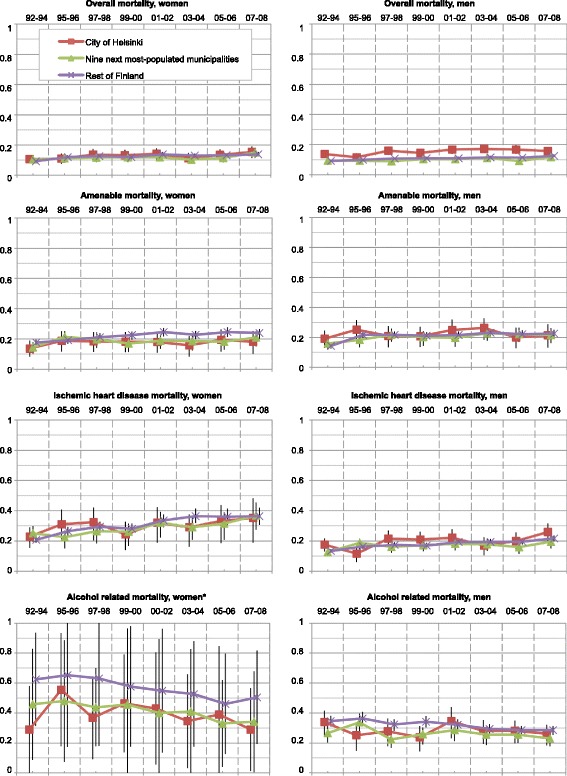
Geographical inequality of mortality distribution between small areas in Finland measured with the Gini coefficient. (*) No bootstrap method was applied in alcohol-related mortality of women

**Fig. 7:**
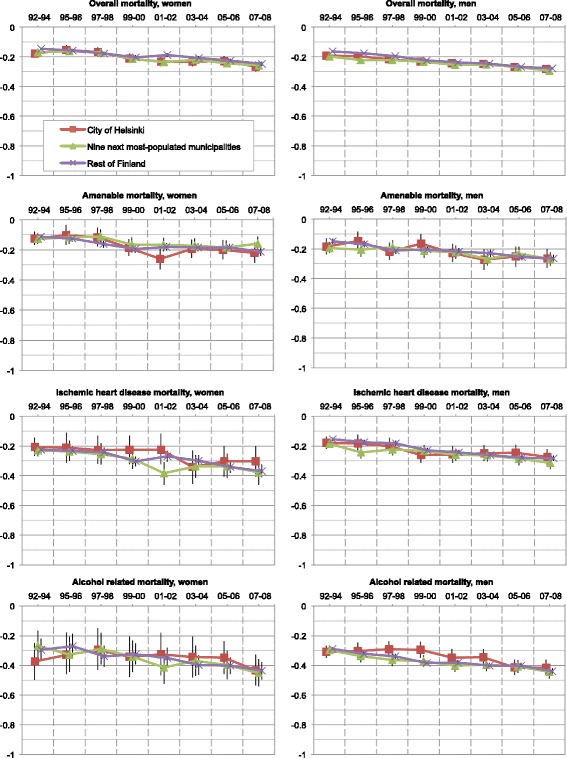
Inequality of mortality distribution between socioeconomic quintiles in Finland measured with Concentration index

While the mortality rates of the working-aged population mainly decreased, the geographical inequalities, measured with Gini coefficients, slowly but steadily increased in all except the alcohol-related mortality (Fig. 6). The excess in overall mortality of men in Helsinki persisted throughout the study period, but diminished with income standardization, suggesting that it arose from socioeconomic disparities. Cs in every geographical area and type of mortality steadily decreased (Fig. 7). As a whole, while the development of Ginis varied — their significance mostly diminished by the income standardization — the annual Cs significantly decreased (Table 4).

**Table 4:** Linear trends for annual Gini coefficients and concentration indices in 1992–2008 in Finland

Mortality	GINI/C	City of Helsinki				Nine next most-populated municipalities				Rest of Finland			
		Women		Men		Women		Men		Women		Men	
		Estimate	95 % Cl	Estimate	95 % Cl	Estimate	95 % Cl	Estimate	95 % Cl	Estimate	95 % Cl	Estimate	95 % Cl
OM	GINI	.0026*^/^ns	.0006 to .0045	.0026**^/^ns	.0010 to .0041	.0014	-.0005 to .0033	.0013*^/^ns	.0001 to .0026	.0024*	.0007 to .0042	.0020**^/^ns	.0005 to .0035
OM	C	-.0061***	-.0078 to -.0044	-.0065***	-.0072 to -.0059	-.0074***	-.0098 to -.0051	-.0061***	-.0070 to -.0051	-.0066***	-.0086 to -.0047	-.0086***	-.0095 to -.0078
AM	GINI	.0020	-.0008 to .0048	.0010	-.0025 to .0045	.0011	-.0020 to .0042	.0038*	.0002 to .0075	.0047**^/^*	.0016 to .0078	.0044*	.0008 to .0080
AM	C	-.0076***	-.0108 to -.0044	-.0085**	-.0134 to -.0036	-.0041*	-.0074 to -.0009	-.0059*	-.0110 to -.0007	-.007***	-.0101 to -.0039	-.0078***	-.0112 to -.0044
IHD	GINI	.0062**	.0017 to .0107	.0045*^/^ns	.0007 to .0082	.0080**^/^***	.0037 to .0123	.0026	-.0012 to .0064	.0114***	.0069 to .0158	.0051**	.0015 to .0087
IHD	C	-.0088**	-.0147 to -.0030	-.0058**	-.0092 to -.0025	-.0105***	-.0141 to -.0069	-.0058***	-.0081 to -.0035	-.0104***	-.0135 to -.0073	-.0094***	-.0121 to -.0068
AR	GINI	-.0050	-.0141 to .0042	-.0019 ns/*	-.0070 to .0033	-.0103*	-.0198 to -.0007	-.0029	-.0076 to .0017	-.0122*/**	-.0217 to -.0027	-.0055*/**	-.0106 to -.0004
AR	C	-.0063*	-.0116 to -.0009	-.0087***	-.0113 to -.0062	-.0101***	-.0151 to -.0052	-.0088***	-.0118 to -.0058	-.0097***	-.0139 to -.0054	-.0103***	-.0128 to -.0078

*OM* overall mortality, *AM* amenable mortality, *IHD* ischemic heart disease mortality, *AR* alcohol related mortality, *GINI* Gini coefficients, markings after slash (/) indicate changed levels of significance due to income standardization, *C* concentration indices, *ns* not significant

*p ≤ .05, **p ≤ .01, ***p ≤ .001

## Discussion

Our results of increasing Ginis (Fig. 6) and decreasing Cs (Fig. 7 and Table 4) showed that both geographical and socioeconomic health inequalities increased in Finland from 1992 until 2008. For example, while mortality in the whole population aged 25–74 decreased, its polarization increased (Fig. 1). Rates of the highest income quintiles developed alike in every geographical area and type of mortality. However, inspection of the Ginis and their development revealed no difference in the segregation of mortality between the City of Helsinki and the other parts of the country. Thus, no definite metropolitan effect emerged in the analyses. The increase in socioeconomic inequalities in favour of the higher income quintiles surpassed the increase of geographical inequalities in every type of geographical area and mortality. These findings suggest that Helsinki, with its policies of positive discrimination and social mixing in town planning and municipal housing, experienced only minor effects of increasing residential differentiation on its mortality. While no other argument supported the existence of independent geographical inequalities, some inequalities occurred even after income standardization.

This observational study compared the geographical variation of mortality rates and compared it against information on residential differentiation in Helsinki from previous studies. The usage of other measures of segregation might have strengthened the link between residential differentiation and its effects on health. In Helsinki, social mixing in town planning and municipal housing disperses the deprived population and its mortality between neighbourhoods and even city blocks. Thus this policy has potentially equalized geographical inequalities in Helsinki in our study; a thorough analysis of neighbourhoods could provide more precise results. Since the population of Helsinki is about a third of the combined population in the nine next most-populated municipalities, and a sixth of the rest of Finland, there was larger variation in the confidence intervals in Helsinki.

While the income quintile division included the total population, the exclusion of the population under age 25 and over 74 affected the size of the analysed quintiles in bigger municipalities, especially in Helsinki with its young and wealthy population: over a third of the population analysed in Helsinki and over a quarter in the nine next most-populated municipalities counted among the highest quintile. As the major portion of mortality is concentrated in the older population rather than in youth, we assume that the chosen age limit decreased the relative overall mortality rates of the lower quintiles in urban areas, thus possibly diluting some of our results. Such a division, however, enabled comparisons of income quintiles between the three geographical areas.

The usage of mortality as an endpoint captured only the most serious illnesses, and thus possibly diluted the existing health inequalities; on the other hand, this prevented any risk of overestimation [41]. Changes in population health can influence mortality with a delay of several years or even decades, thus necessitating our follow-up of 17 years. Applicable mortality data existed even before the chosen study period, but the unavailability of income data prevented an extension of the time frame.

Finnish mortality and income-level data are in general of good quality and reliable throughout the relatively long study period, thus enabling the linkage of individual incomes and small-area-level composition [42, 43]. This allowed for an evaluation of the development of health inequalities over time. Our usage of aggregated data, Ginis and Cs enabled the analysis even while the level of mortality in some small areas was too low for direct mortality comparisons.

### Overall and alcohol-related mortality

Almost all geographical differences in overall mortality disappeared after income standardization, which points to increasing socioeconomic inequalities. The nationwide inequality increased, as the mortality rates in the lowest income quintile remained stagnant, while declining in others. This corresponds with the previous findings, which describe a slow increase in life expectancy within this population group [2, 29, 38, 44]. Surprisingly alcohol-related mortality in the other parts of Finland, especially in the lowest quintile, caught up with Helsinki, indicating that the use of alcohol has increased in small areas with a history of lower alcohol mortality. This development accelerated after 2004 due to the reduction in excise tax on alcohol [45]. Our observations support the interpretation that the increased use of alcohol in the lowest income quintile accounts for a significant proportion of Finland’s increasing socioeconomic inequalities in mortality [38, 46].

For still unknown reasons, the mortality of the second lowest income quintile in Helsinki remained elevated and overlapped the mortality of the lowest quintile. This finding differs from other parts of the country, both urban and rural. Previous research has yielded similar findings: although overall morbidity is lower in Helsinki than elsewhere in Finland, life expectancy is paradoxically shorter [2]. Findings from Lisbon suggest that urbanization level reduces the effect of material deprivation on mortality [47], which could decrease mortality in the lowest quintile. Another possible hypothesis may, for instance, be that the burden of a higher cost of living in Helsinki extends to the second lowest quintile, especially among those living alone. This burden potentially hinders social mobility, social capital, and sustaining of social networks, thus increasing the risk of social exclusion, and eventually exposing individuals to poor health [48–50]. The equivalent effect that living alone had on the risk ratios for mortality in men of the lowest quintiles (Q1–Q2) in Helsinki supports this hypothesis.

### Amenable and IHD mortality

In amenable mortality there was practically no significant increase in geographical inequalities; in IHD mortality, however, inequalities increased mainly in women throughout the country. None of the three geographical areas analysed stood out from the others. The low mortality rates of IHD in women accounted for its uneven geographical distribution and inequality between the small areas. People in remote locations are at risk of treatment delays, and thus prone to serious complications. According to Henriksson et al., the risk for acute myocardial infarction (AMI) in Sweden is low in urban small areas with high levels of income inequality, mostly due to the decreased risk for non-manual workers [18]. Additionally, they propose that high levels of regional segregation and income inequality are associated with high levels of health inequalities. We included no assessment of AMIs alone in the analyses. No similar trend, however, emerged in our study. When considering socioeconomic inequalities, both types of mortality increased alike throughout the different types of geographical areas and income quintiles.

It has been suggested that the increase in relative inequalities of amenable mortality in Finland results from socioeconomic inequity in access to and treatment in health care [36]. Our findings supported this suggestion as socioeconomic inequalities surpassed geographical ones. In addition, a corresponding decrease of absolute mortality and an increase of relative inequalities existed in overall and IHD mortality. Our findings differed from the urban areas of Spain, where socioeconomic inequalities of preventable mortality remained stable [51].

Previous studies question the use of avoidable mortality as an indicator of inequality for both the quality of and access to health care between income groups. No strong associations have been found between changes in either mortality for specific amenable conditions and the introduction of specific health care innovations or inequalities in mortality and access to or quality of health care - thus caution is advised when interpreting the results [52, 53]. We analysed national amenable mortality rates over time, and evaluated both its geographical and socioeconomic development in parallel with the other types of mortality. Thus, we took into account the suggested caution.

### Residential differentiation

In spite of knowledge of increasing residential differentiation in the capital, amenable and other types of mortality in Helsinki varied similarly to mortality in other parts of the country. In lower income quintiles the mortality rates in Helsinki were slightly elevated, however. Our findings for residential differentiation and the geographical distribution of mortality suggest a couple of possible interpretations: 1) despite the long time frame studied, it is possible that the time-lag between differentiation and mortality is longer than expected, and 2) the level of differentiation between Helsinki and the other geographical areas analysed was more similar than presumed (possibly due to positive discrimination and social mixing in town planning and municipal housing in Helsinki). Although the differentiation in Helsinki Metropolitan Area evidently increases [26], a countrywide assessment of development of differentiation is needed to test these interpretations.

## Conclusions

Our comparative analyses revealed no differences in the geographical or socioeconomic distribution of mortality between the capital city, other urban areas, and rural parts of Finland. Overall, amenable, and ischemic heart disease mortalities decreased alike throughout Finland. Alcohol-related mortality was one of the main culprits for increasing health inequalities, and its growth showed no signs of slowing down. It would be prudent to find ways to tackle this development before even greater differences arise. Thus our hypothesis of the ‘metropolitan effect’, i.e. increasing geographical inequalities in the City of Helsinki that would be unseen elsewhere in Finland, was not supported. This may relate to the policies of positive discrimination and social mixing in Helsinki. The increase in socioeconomic inequalities surpassed the increase in geographical ones throughout the country: the higher the income quintile, the more the people in it benefited from the development of society and health care.
